# A novel microdeletion of 517 kb downstream of the *PAX6* gene in a Chinese family with congenital aniridia

**DOI:** 10.1186/s12886-023-03147-1

**Published:** 2023-09-26

**Authors:** Yinwen Li, Jieqiong Chen, Ying Zheng, Zhixuan Chen, Tao Wang, Qian Sun, Xiaoling Wan, Haiyun Liu, Xiaodong Sun

**Affiliations:** 1 Department of Ophthalmology, Shanghai General Hospital (Shanghai First People’s Hospital), Shanghai Jiao Tong University, School of Medicine, Shanghai, China; 2 National Clinical Research Center for Eye Diseases, Shanghai, China; 3 Shanghai Key Laboratory of Ocular Fundus Diseases, Shanghai, China; 4 Shanghai Engineering Center for Visual Science and Photomedicine, Shanghai, China

**Keywords:** Aniridia, *PAX6*, Copy number variant, Microdeletion, Regulatory elements

## Abstract

**Background:**

To identify the disease-causing gene in a Chinese family affected with congenital aniridia.

**Methods:**

Patients underwent systematic ophthalmic examinations such as anterior segment photography, fundus photography, optical coherence tomography, and fundus fluorescein angiography. The proband was screened for pathogenic variants by whole exome sequencing (WES) and copy number variant (CNV) analysis. Real-time quantitative PCR (RT-qPCR) was applied to confirm the CNV results. Breakpoints were identified by long-range PCR followed by Sanger sequencing.

**Results:**

All seven members of this Chinese family, including four patients and three normal individuals, were recruited for this study. All patients showed bilateral congenital aniridia with nystagmus, except the son of the proband, who presented with bilateral partial coloboma of the iris. A novel heterozygous deletion (chr11:31,139,019–31,655,997) containing the 3’ regulatory enhancers of the *PAX6* gene was detected in this family. We also reviewed the reported microdeletions downstream of *PAX6* in patients with aniridia.

**Conclusions:**

We identified a novel microdeletion, 517 kb in size located about 133 kb downstream of the *PAX6* gene, responsible for congenital aniridia in this Chinese family, which expands the spectrum of aniridia-associated mutations in *PAX6*.

**Supplementary Information:**

The online version contains supplementary material available at 10.1186/s12886-023-03147-1.

## Background

Aniridia (OMIM, 106210) is a rare, bilateral, congenital panocular disorder characterized by the partial or complete absence of the iris, with an incidence of approximately one in 40,000–100,000 live births worldwide. About two-thirds of aniridia cases are familial with autosomal dominant inheritance, while the remaining cases are sporadic [1]. In addition to iris hypoplasia appearing in early infancy, congenital aniridia is usually associated with aniridia-associated keratopathy, glaucoma and cataract, resulting in severely reduced vision in the patient’s second or third decade [2]. Aniridia can also occur as part of other syndromes such as WAGR (OMIM, 194072) [3, 4], WAGRO (OMIM, 612469) [5], Peters anomaly (OMIM, 604229) [6, 7], and Gillespie syndrome (OMIM, 206700) [8, 9].

Paired box gene-6 (encoded by *PAX6*, OMIM, 607108) is a highly conserved transcriptional regulator that plays a key role in normal ocular and neural development [6]. It is 22 kb in size and locates on chromosome 11p13, but has regulatory regions spanning ~ 450 kb of genomic DNA [10]. Heterozygous mutations in the *PAX6* gene or its regulatory regions cause aniridia [11, 12]. To date, more than 600 variants scattered throughout *PAX6* locus have been identified, according to the *PAX6* gene database (LOVD, https://www.lovd.nl/). Of note, chromosomal rearrangements in the 11p13 region are frequently identified in aniridia cases, and these are thought to affect 3′ regulatory enhancers of the *PAX6* gene and influence the expression level of the PAX6 protein [13].

In this study, we identified a novel microdeletion with a length of 517 kb downstream of the *PAX6* gene in a Chinese family with congenital aniridia, although two copies of *PAX6* are intact. This 11p13 microdeletion contained essential conserved enhancer elements of *PAX6*, which was likely to be the cause of the familial aniridia in this family.

## Methods

### Subject recruitment and clinical assessment

**Fig. 1:**
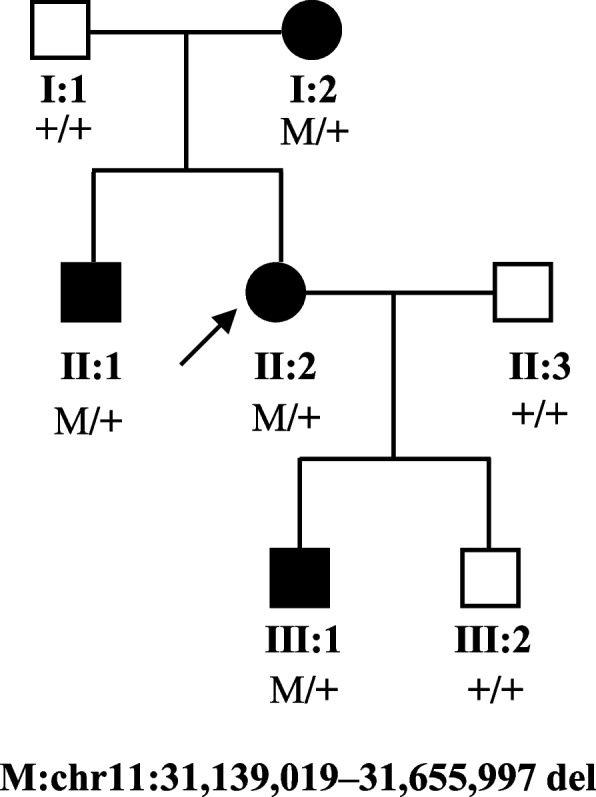
Pedigree of the three-generation family with congenital aniridia. Squares and circles indicate males and females respectively. Solid symbols indicate affected individuals, and open symbols indicate unaffected individuals. The arrow indicates the proband of this family

This study was conducted in accordance with the Declaration of Helsinki and with the approval of the medical ethics committee of Shanghai General Hospital, China (Approval No.2020SQ328). A three-generation family with aniridia was recruited and seven family members of this family (Fig. 1) participated in this study. All research subjects provided informed consent. Three of the seven family members were diagnosed with congenital aniridia, and one was with iris coloboma. There was no consanguinity present in this family. Complete and comprehensive clinical and ophthalmic examinations were conducted for each participant, including best corrected visual acuity (BCVA) by Snellen visual acuity, non-contact tonometer, non-contact intraocular pressure (IOP) measurement (TX-20, Canon), slit lamp biomicroscopy, anterior segment photography, fundus photography (200TX, OPTOS), optical coherence tomography (Spectralis, Heidelberg Engineering), fundus fluorescein angiography (Optomap plus, OPTOS), and indocyanine green angiography.

### Whole-exome sequencing (WES)

The WES of the gDNA samples was performed as previously described [14, 15]. The genomic DNA was extracted from peripheral blood samples of the members (Qiagen GmbH, Hilden, Germany). The DNA fragments were hybridized and captured by IDT’s xGen Exome Research Panel V1.0 (Integrated DNA Technologies, San Diego, USA) according to the manufacturer’s Protocol. Novaseq.6000 platform (Illumina, San Diego, USA), with 150 bp pair-end sequencing mode, was used for sequencing the genomic DNA of the proband. Raw image files were processed using CASAVA v1.82 for base calling and generating raw data. The raw reads were aligned to the human reference genome (GRCh38/hg38) using the Burrows Wheeler Aligner tool. Duplicates of the reads were marked and removed using Picard (http://broadinstitute.github.io/picard).

Single-nucleotide variants (SNVs) and InDels (Insertions and Deletions) were called by GATK4-HaplotypeCaller. The frequencies of all SNVs and InDels are annotated using Genome Aggregation Database (gnomAD), the 1000 Genome project (http://brower.1000genomes.org), and the China Metabolic Analytics Project (ChinaMAP) to filter the common variants, with an allele frequency cutoff of 5%. Pathogenicity of remaining variants is predicted by proper analytic tools, including PolyPhen-2 (http://genetics.bwh.harvard.edu/pph2/) and SIFT (http://sift.jcvi.org/).

For CNV analysis, we applied eXome-Hidden Markov Model (XHMM, zzz.bwh.harvard.edu/xhmm/) principal component analysis (PCA) to normalize the read depth and remove the sequencing noise, and used CNVKit (www.github.com/etal/cnvkit) to perform GC and bias correction. CNV calculation and identification were performed by XHMM Hidden Markov Model (HMM). Variant and CNV filtering was performed as illustrated in Supplementary Figure 1. Detailed information of WES coverage and sequencing depth was listed in Supplementary Table 1.

### Copy number variant (CNV) validation by real-time quantitative PCR and breakpoints identification by long-range PCR

CNVs were further validated by real-time quantitative PCR (RT-qPCR). RT-qPCR was accomplished using the SYBR Premix Ex Taq (Takara, Japan) in the ABI PRISM® 7300 real-time-PCR system (Applied Biosystem, Foster City, CA, USA). GAPDH was used as an endogenous control. The relative copy number was computed using 2^−ΔΔCt^ method. Then to define the breakpoint, fragments surrounding the breakpoints were amplified by long-range PCR and Sanger sequencing (Huagene, Shanghai, China). The sequences of the primers are listed in Supplementary Table 2. To define the genomic architecture in triggering genomic rearrangement events, upstream and downstream regions around the breakpoint were analyzed using the RepeatMasker track (http://www.repeatmasker.org/) of UCSC Genome Browser (https://genome.ucsc.edu/).

## Results

### Clinical findings

**Fig. 2:**
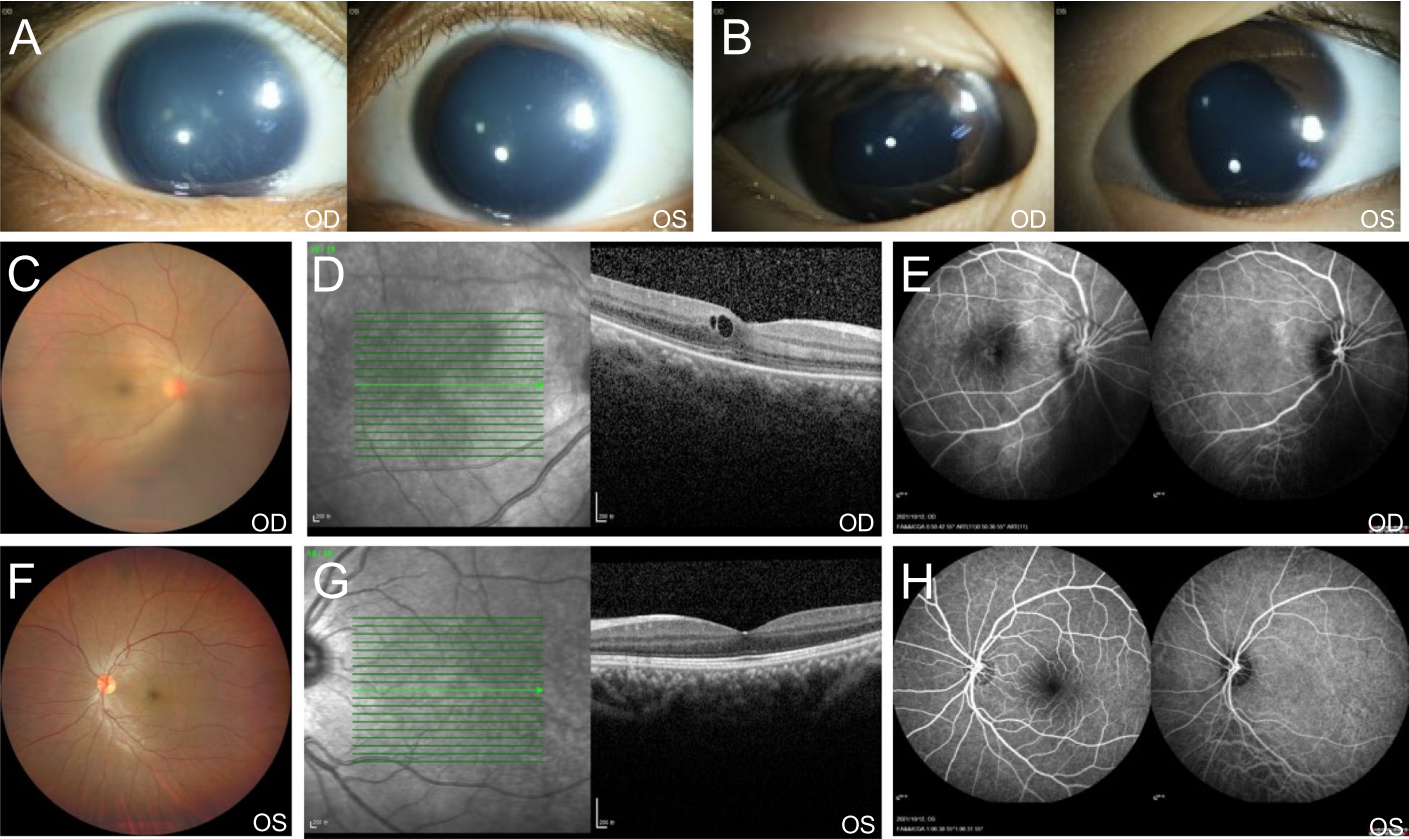
Representative photos of ophthalmological findings. **A** Anterior segment photograph of the proband (II:2) performed aniridia with peripheral iris remnants in both eyes. **B** Anterior segment photograph of the patient (III:1) displayed bilateral partial coloboma of iris. **C**, **F** No obvious abnormalities were found in the fundus images of the proband except mild vitreous opacity in the right eye. **D**, **G** Optical coherence tomography (OCT) of the proband displayed cystoid macular edema in the right eye. **E**, **H** Fundus angiography of the proband showed slight leakage in the right macular and the optic disc. OD, oculus dexter; OS, oculus sinister

In this study, we identified a three-generation family with congenital aniridia including four affected members, as shown in Fig. 1. All three adult-affected patients (I:2, II:1, and II:2) presented with low visual acuity, ranging from 0.2 to 0.3. The Intraocular pressure of the three adult patients was normal (I:2, II:1, and II:2). These three adult patients (I:2, II:1, and II:2) showed phenotypes of bilateral aniridia and nystagmus, while one patient (III:1) was found to suffer from bilateral partial coloboma of the iris (Table 1). Representative photos from anterior segment photography of II:2 (proband) and III:1 are shown in Fig. 2. Mild vitreous opacity, cystoid macular edema, and leakage were observed in the right eye of the proband (Fig. 2). No other ophthalmic phenotypes, such as foveal hypoplasia, microphthalmia, lens defects, and non-ocular abnormalities, such as mental retardation, emotional disorders, renal diseases, and neurological deficits were detected in the four patients. No other family members had aniridia phenotypes or other relevant eye diseases.

**Table 1:** Clinical characteristics of the four patients in this Chinese family

Patients	Age/year	Gender	Eye	BCVA	IOP/mmHg	Iris
I:2	55	F	OD	0.3	17.3	Aniridia
			OS	0.3	16.6	Aniridia
II:1	33	M	OD	0.3	15.7	Aniridia
			OS	0.3	15.4	Aniridia
II:2	29	F	OD	0.2	14.9	Aniridia
			OS	0.2	16.7	Aniridia
III:1	4	M	OD	NA	NA	Coloboma
			OS	NA	NA	Coloboma

Other ophthalmologic findings including nystagmus, keratopathy, cataract, and glaucoma were absent in all patients in the family

*M* male, *F* female, *OD* the right eye, *OS* the left eye, *BCVA* best corrected visual acuity, *IOP* intraocular pressure, *NA* not available

### Genetic analysis

To determine the disease-causing gene in this family, whole exome sequencing (WES) was performed on genomic DNA from the peripheral blood of the proband. No possible pathogenic single nucleotide variants (SNVs) and indels related to aniridia were detected in the proband, according to the guidelines of the American College of Medical Genetics and Genomics (ACMG) [16]. However, a possible pathogenic copy number variant (CNV) was detected by XHMM V1.0. A heterozygous deletion of approximately 410 kb (chr11:31,241,412–31,650,221) was found, which covered adjacent genes at the 3’ end of *PAX6* including exons 1–9 of doublecortin domain containing 1 (*DCDC1*), DnaJ heat shock protein family (Hsp40) member C24 (*DNAJC24*), inner mitochondrial membrane peptidase subunit 1 (*IMMP1L*), and exons 1–9 of elongator acetyltransferase complex subunit 4 (*ELP4*).

### Identification of a CNV in this family

**Fig. 3:**
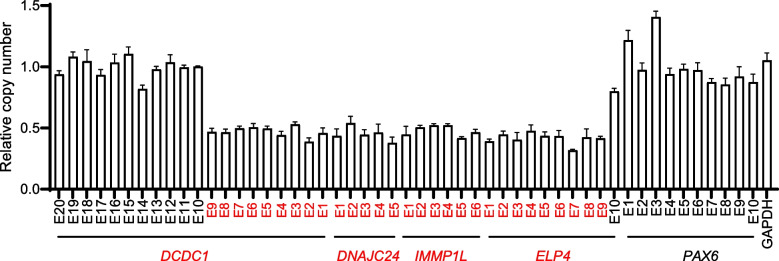
CNV confirmation by RT-qPCR in the proband of this Family. The relative copy number of exons 1 − 20 of *DCDC1*, *DNAJC24*, *IMMP1L*, *ELP4*, and *PAX6* in the proband of this Family. The affected exons and genes were colored in red

To confirm the CNV detected by WES in this family, we performed real-time quantitative PCR (RT-qPCR) to examine the copy number of affected exons. The relative copy number of the exons 1–9 of *DCDC1*, *DNAJC24*, *IMMP1L*, and exons 1–9 of *ELP4* in the proband was half that in a healthy control, which is consistent with the results of WES (Fig. 3).

**Fig. 4:**
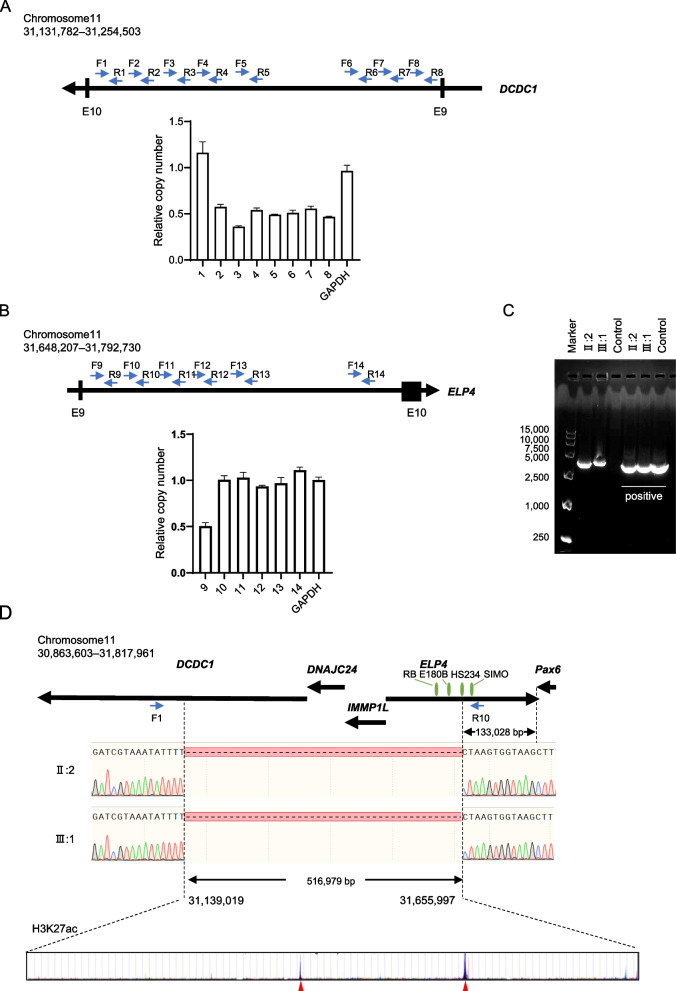
Identification of the breakpoints of CNV in this family. **A** The relative copy number of different regions in intron 9 of *DCDC1*. **B** The relative copy number of different regions in intron 9 of *ELP4*. **C** Long-range PCR by primer pair F1/R10 on gDNA from the proband. An approximate 3,000 bp fragment was amplified in II:2 and III:1, but not in a healthy control. **D** Sequence chromatograms showing the microdeletion (chr11:31,139,019–31655997) (hg38) identified in this study

The sizes of intron 9 of *DCDC1* and intron 9 of *ELP4* are both longer than 100 kb, which are too large to amplify by long-range PCR. To further define the breakpoints of the CNV, we used RT-qPCR to identify the deleted regions in intron 9 of *DCDC1* and intron 9 of *ELP4* (Fig. 4A and B). Then, we performed long-range PCR with primers F1 and R10. An approximately 3,000 bp fragment was amplified in the genomic DNA from the proband (II:2) and her son (III:1), but not from a healthy control (Fig. 4C). Original gels are presented in Supplementary Figure 2. Sanger sequencing of the PCR products identified a 516,979 bp deletion (chr11:31,139,019–31,655,997) between the proximal and distal breakpoints in II:2 and III:1. The proximal breakpoint of this microdeletion was approximately 123 kb from the 3’ end of the *PAX6* and was located in the HS234 fragment (hypersensitive sites 2, 3, and 4) (chr11: 31,652,512–31,657,084) in intron 9 of *ELP4*. This microdeletion involved three conserved regulatory elements (RB, E180B, and HS) of *PAX6* and two H3K27ac enrichment sites (Fig. 4D).

To investigate whether repetitive elements intersected with the breakpoints, which may lead to genomic instability and CNVs [17], we used RepeatMasker to align the breakpoints with specific repetitive elements. Unfortunately, the breakpoints were located in regions without repeated elements, indicating that other mechanisms may be involved in the genomic arrangement in this family. Taken together, a 517 kb heterozygous deletion containing four annotated genes, *DCDC1*, *DNAJC24*, *IMMP1L*, and *ELP4*, was identified in this Chinese family with congenital aniridia, suggesting that transcription-regulating elements in the deleted region are required for the expression of PAX6.

### Overview of previously reported microdeletions excluding the *PAX6* gene

**Fig. 5:**
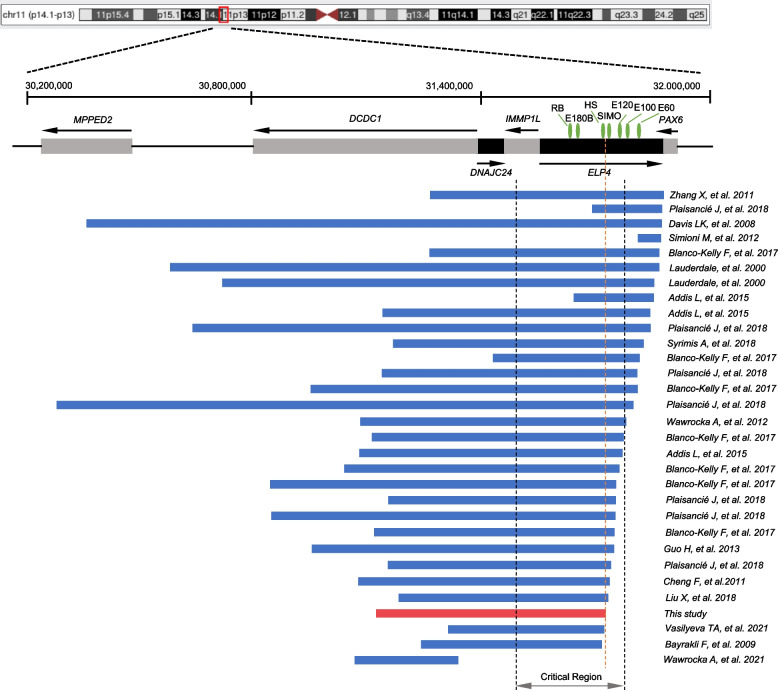
Schematic diagram of 11p13 microdeletions involving regulatory regions 3’ of *PAX6* identified in patients with aniridia. Genes are represented by grey and black boxes and arrows indicate the direction of transcription. Known *PAX6* enhancers, RB, E180B, HS, SIMO, E120, E100, and E60 are indicated by green oval shapes. Horizontal blue bars represent microdeletions in 11p13 identified in aniridia patients with the integrity of *PAX6*. The vertical dashed lines indicated the “critical region” required for the *Pax6* gene transcription, which was defined first as 244 kb long by Ansari et al. Genomic coordinates are based on human genome assembly hg38

Microdeletions involving the 11p3q region are frequent in cases with congenital aniridia, and these microdeletions partly or completely encompass the *PAX6* gene or remove the *PAX6* gene. [2, 4, 13]. To date, 30 genomic rearrangements downstream of the *PAX6* have been reported in the literature to cause aniridia without neurodevelopment disorders (Fig. 5 and Supplementary Table 3) [18–31]. These deleted regions range from 49 to 1,300 kb, and the distance from the 3’ of *PAX6* range from 1 to 467 kb. Among them, 29 microdeletions entirely or partially spanned the critical region, which is required for the *PAX6* gene transcription, as suggested by Ansari et al. 2016 [9].

## Discussion

Congenital aniridia is a severe bilateral panocular visual disorder with an autosomal dominant inheritance. *PAX6*, a highly conserved transcription factor, plays a fundamental role in organogenesis by regulating downstream target genes [6, 32, 33]. Many studies [34–39] have reported that mutations in the *PAX6* gene and its regulatory regions are the leading cause of congenital aniridia. In this study, the novel 517 kb heterozygous deletion (chr11:31,139,019–31,655,997) downstream of *PAX6* containing four annotated genes, *DCDC1*, *DNAJC24*, *IMMP1L*, and *ELP4*, is likely to be the cause of the familial aniridia in this Chinese family. This finding further demonstrates the importance of 3’ downstream regulatory elements on the expression of *PAX6* and broadens the spectrum of *PAX6* mutations resulting in congenital aniridia.

Defects in *PAX6* lead to a broad range of clinical phenotypes, with the most common being aniridia. In addition to varying degrees of iris defects, other associated ocular phenotypes (e.g. microphthalmia, optic nerve anomalies, and anterior segment dysgenesis) [40, 41] and systemic abnormalities (e.g. hormonal, metabolic, gastrointestinal, genitourinary, and neurologic disorders) [3–9] have been reported in some cases with *PAX6* mutations. Moreover, disruption of nearby genes (*ELP4*, *IMMP1L*, *DCDC1*, and *DNAJC24*) may contribute to a range of neurodevelopmental phenotypes [19, 26, 42, 43]. However, in this three-generation family, no other obvious ocular or non-ocular abnormalities were observed in the four patients with congenital aniridia, which is consistent with previous studies showing that defects in the 3’-cis-regulatory regions are generally associated with a milder phenotype without keratopathy, nystagmus, or foveal hypoplasia [26, 30]. Of note, attention should be paid to mild iridopathy in III:1, which may lead to potential blindness and more serious phenotypes in later generations.

*PAX6* is known to be affected by a downstream cluster of ultraconserved transcriptional regulatory elements, such as RB, E180B, HS, SIMO, E100, and E60, which play roles as important enhancers in regulating normal PAX6 expression by transgenic reporter studies [29, 44]. RB, the most distal *PAX6* enhancer, contributes to its expression in prosomere P2 and the pineal gland [45]. E180B drives the expression in the trigeminal nerves and spinal nerve tracts [46]. DNase hypersensitivity cluster (HS1-8), a marker for active cis-regulatory sequences, is located 130 kb downstream of the *PAX6* poly(A) site [47, 48]. Deletion of HS6 results in a complete loss of expression in the pre-cerebellar neuroepithelium, pontine migratory streams, and pre-cerebellar nuclei. Separating of HS5 abolishes expression in the lateral band around the thalamus. HS234, a ~ 4 kb fragment, recapitulated distinct parts of the endogenous expression pattern of PAX6 [44]. Therefore, the defects of RB, E180B, and HS elements are likely to be the underlying cause of familial aniridia by affecting the PAX6 expression in this Chinese family. Further studies are needed to verify their transcription-regulating capability of PAX6.

## Conclusion

In conclusion, a novel 517 kb heterozygous deletion of chromosome 11p13 downstream of the *PAX6* is likely to be the underlying cause of iris abnormalities in this family. These results enrich the mutation spectrum of *PAX6*, providing further evidence that genetic defects in the 3’ regulatory elements downstream region of *PAX6* lead to congenital aniridia.

### Supplementary Information

**Additional file 1:** **Supplementary Figure 1. **Filtering strategy of variants and CNVs. (A) single nucleotide variants/indels; (B) copy number variants.

**Additional file 2:** **Supplementary Figure 2. **Original gels of Long-range PCR in Fig. 4.

**Additional file 3:** **Supplementary Table 1.** Detailed information of WES coverage and sequencing depth.

**Additional file 4:** **Supplementary Table 2**. Primers used for defining the breakpoint.

**Additional file 5:** **Supplementary Table 3**.

## Data Availability

The datasets generated during the current study are available in the Sequence Read Archive (SRA) repository, the accession number is SRR21619456 and the persistent web link is https://www.ncbi.nlm.nih.gov/sra/?term=SRR21619456.

## Abbreviations

PAX6: Paired box gene-6
WES: Whole exome sequencing
CNV: Copy number variant
RT-qPCR: Real-time quantitative PCR
WAGR: Wilms tumor, aniridia, genitourinary anomalies, and mental retardation syndrome
WAGRO: WAGR Syndrome with obesity
OCT: Optical coherence tomography
BCVA: Best corrected visual acuity
IOP: Intraocular pressure
NA: Not available
SNV: Single nucleotide variant
ACMG: American College of Medical Genetics and Genomics
DCDC1: Doublecortin domain containing 1
DNAJC24: DnaJ heat shock protein family (Hsp40) member C24
IMMP1L: Inner mitochondrial membrane peptidase subunit 1
ELP4: Elongator acetyltransferase complex subunit 4
HS: Hypersensitive sites
