# Supplementary material for: A novel microdeletion of 517 kb downstream of the PAX6 gene in a Chinese family with congenital aniridia
Source: BMC Ophthalmol. 2023 Sep 26;23:393. doi: 10.1186/s12886-023-03147-1 (PMC10523764; doi:10.1186/s12886-023-03147-1)

Supplementary Figure 1. Filtering strategy of variants and CNVs. (A) single nucleotide variants/indels; (B) copy number variants.

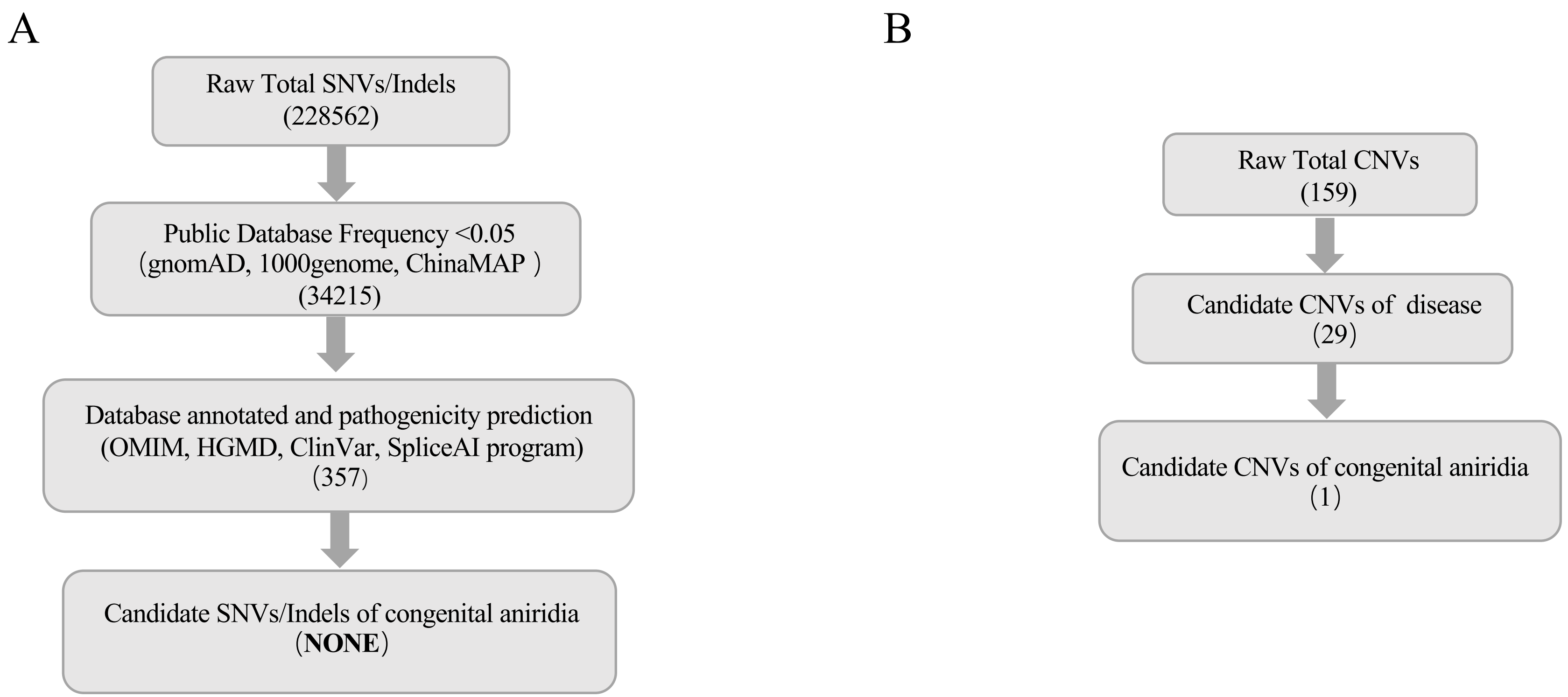

Supplement: Supplementary file 1 — Additional file 1: Supplementary Figure 1. Filtering strategy of variants and CNVs. (A) single nucleotide variants/indels; (B) copy number variants. [file 12886_2023_3147_MOESM1_ESM.pdf]
