# Supplementary figures and images for: A novel microdeletion of 517 kb downstream of the PAX6 gene in a Chinese family with congenital aniridia
Source: BMC Ophthalmol. 2023 Sep 26;23:393. doi: 10.1186/s12886-023-03147-1 (PMC10523764; doi:10.1186/s12886-023-03147-1)

Supplementary Figure 2. Original gels of Long-range PCR in Fig.4

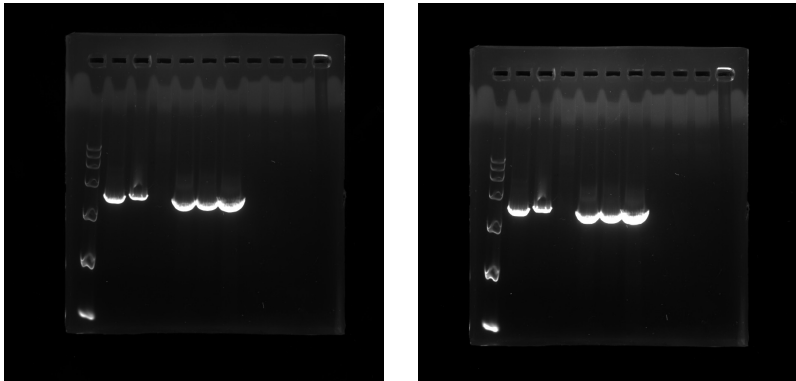

Supplement: Supplementary file 2 — Additional file 2: Supplementary Figure 2. Original gels of Long-range PCR in Fig. 4. [file 12886_2023_3147_MOESM2_ESM.pdf]
