# Supplementary material for: A novel microdeletion of 517 kb downstream of the PAX6 gene in a Chinese family with congenital aniridia
Source: BMC Ophthalmol. 2023 Sep 26;23:393. doi: 10.1186/s12886-023-03147-1 (PMC10523764; doi:10.1186/s12886-023-03147-1)
Supplement: Supplementary file 3 — Additional file 3: Supplementary Table 1. Detailed information of WES coverage and sequencing depth. [file 12886_2023_3147_MOESM3_ESM.docx]

**Supplementary Table 1.** Detailed information of WES coverage and sequencing depth

| Sample | Proband |
| --- | --- |
| Coverage ratio | 2.13% (64M/3GB) |
| Total reads | 51,232,952 |
| Mapped reads | 51,217,130 |
| Mapping rate (%) | 99.97 |
| Mean depth | 93.12 |
| Coverage at least 4X (%) | 99.8 |
| Coverage at least 10X (%) | 99.7 |
| Coverage at least 20X (%) | 99.3 |
| Coverage at least 50X (%) | 87.6 |
