# Supplementary material for: A novel microdeletion of 517 kb downstream of the PAX6 gene in a Chinese family with congenital aniridia
Source: BMC Ophthalmol. 2023 Sep 26;23:393. doi: 10.1186/s12886-023-03147-1 (PMC10523764; doi:10.1186/s12886-023-03147-1)
Supplement: Supplementary file 4 — Additional file 4: Supplementary Table 2. Primers used for defining the breakpoint. [file 12886_2023_3147_MOESM4_ESM.docx]

**Supplementary Table 2**. Primers used for defining the breakpoint.

| Primer name | Sequence (5'-3') | Primer name | Sequence (5'-3') |
| --- | --- | --- | --- |
| DCDC1-E1-F | gaggaggagagaagcgacag | DCDC1-E11-F | atgagaggcacagaacgaca |
| DCDC1-E1-R | gtttctggccgagctgatg | DCDC1-E11-R | ggtgagcaggctgattgatg |
| DCDC1-E2-F | tgtccaaccagtcccaatga | DCDC1-E12-F | tggtcagtttgaattgggct |
| DCDC1-E2-R | tgggcacattgttttggtct | DCDC1-E12-R | acacaattttcagttgacatggt |
| DCDC1-E3-F | cccatccaaagtgccttcag | DCDC1-E13-F | aaaccccttgctcctacctg |
| DCDC1-E3-R | tgtgtttaatgcccagcctc | DCDC1-E13-R | ctatcccctctgcccctcta |
| DCDC1-E4-F | tggctgtgatctgatgctgt | DCDC1-E14-F | acttgccacatccaggtagc |
| DCDC1-E4-R | atgtcatcccaggcaaaagc | DCDC1-E14-R | tgacctttgaccgagtgagt |
| DCDC1-E5-F | aagcacagaaaactttggaaca | DCDC1-E15-F | aatcccaccttgttctgtagaa |
| DCDC1-E5-R | tttcagtcagcccggaattg | DCDC1-E15-R | agttgggaagtttgtgaggga |
| DCDC1-E6-F | gcaccccttaagcaattcagt | DCDC1-E16-F | ggtcactcagcaaaagcaga |
| DCDC1-E6-R | ggaggagtgcacagaaaagc | DCDC1-E16-R | gtccttcatgcctctgtttcc |
| DCDC1-E7-F | cccactgtaatctcatgccc | DCDC1-E17-F | ctccctaacaggcactgagc |
| DCDC1-E7-R | tgaatgggttgatgcttcct | DCDC1-E17-R | tcttttggccctgtaccaac |
| DCDC1-E8-F | tctttgccacttaccttttgaaa | DCDC1-E18-F | ccatcagttccaaacaccca |
| DCDC1-E8-R | cacacatgcaagtacaatgtagc | DCDC1-E18-R | tttggtagagtgtgggcagt |
| DCDC1-E9-F | caggaggcatcacaattgaa | DCDC1-E19-F | gccatgttcctgatgagctg |
| DCDC1-E9-R | ctgcctggctattggtcatc | DCDC1-E19-R | tcctcctctctttccagttgg |
| DCDC1-E10-F | ctggccctcattgcagtttt | DCDC1-E20-F | cagtaccctacctctgagcag |
| DCDC1-E10-R | agatacctgctttcatgctgt | DCDC1-E20-R | agaaagtgccagcaaccttg |
| DNAJC24-E1-F | agcagcgcctatgtgaagtt | PAX6-E1-F | tgctgctgttgttgcttgaa |
| DNAJC24-E1-R | ccacgctccagaagagagac | PAX6-E1-R | attgctctcacacaccaacc |
| DNAJC24-E2-F | agatacatggcaaactgtgatga | PAX6-E2-F | ctgaaatctcggatgtctgt |
| DNAJC24-E2-R | atctgctcaaccgccatcat | PAX6-E2-R | ggggaagactttaactaggg |
| DNAJC24-E3-F | accccactcagctctctcta | PAX6-E3-F | gttctgcatgctggctctgg |
| DNAJC24-E3-R | cgctgcaggtcatactctct | PAX6-E3-R | gctaacagagccccatattc |
| DNAJC24-E4-F | aggaccagtagatgctcaagt | PAX6-E4-F | tgcagaattcgggaaatgtcg |
| DNAJC24-E4-R | ccagacagaaacacttaccaca | PAX6-E4-R | gtcacagcggagtgaatcag |
| DNAJC24-E5-F | ggtagtcatccaggttcccc | PAX6-E5-F | caaagatggacgggcactc |
| DNAJC24-E5-R | ctcccgttgaaaccgtgtac | PAX6-E5-R | tccaacggatgtgtgagtaaa |
| IMMP1L-E1-F | caaagaaccctggagaccct | PAX6-E6-F | gaagtccccggataccaacc |
| IMMP1L-E1-R | aactctgggaacgcaaacac | PAX6-E6-R | gttcttcgcaacctggctag |
| IMMP1L-E2-F | aacacactttccttccatttgaa | PAX6-E7-F | ctccagggcctcaatttgc |
| IMMP1L-E2-R | cttcgtggtgttctggggaa | PAX6-E7-R | tgccagcaacaggaaggag |
| IMMP1L-E3-F | ggaactgcagaaaagccaca | PAX6-E8-F | ctgtattcttgcttcaggt |
| IMMP1L-E3-R | catcaatggagcctacaattcaa | PAX6-E8-R | agtttgagagaacccattatc |
| IMMP1L-E4-F | ttttcttttgtcagcactcagtt | PAX6-E9-F | gtgtggtgggttgtggaatt |
| IMMP1L-E4-R | tcagaggtgacattgtgattgc | PAX6-E9-R | aggaatcagagaagacaggcc |
| IMMP1L-E5-F | agtacactgacagcttgaagat | PAX6-E10-F | atttgccatggtgaagctgg |
| IMMP1L-E5-R | tgggtcatgtttggttagaagg | PAX6-E10-R | ttcctccttcacatctggct |
| IMMP1L-E6-F | tgaactaacagaacttggcaga | F1 | tcattttccaaggccacttc |
| IMMP1L-E6-R | tttacgtgccagccctaatg | R1 | ggaaactttgggaaatgcaa |
| ELP4-E1-F | actggaggctctaagatggc | F2 | gaatttggccaacaagcagt |
| ELP4-E1-R | ttgataccagcagctgtcca | R2 | acctaggcttccatgtgtgg |
| ELP4-E2-F | gtggaggtttagccgttgg | F3 | atgtagccccttgtgaccat |
| ELP4-E2-R | agcatgtattttgtactaac | R3 | tgggatcactgctgttctga |
| ELP4-E3-F | gccatttgattcttttcttgc | F4 | ttgctctgttgtccaggcta |
| ELP4-E3-R | ttggcaggatcctctttagc | R4 | ggtgaaaccccgtctctaca |
| ELP4-E4-F | acttccagcaccattacttga | F5 | ttcatagggcaaatgcttcc |
| ELP4-E4-R | gtaacgccaagctattttcatct | R5 | cttactgcctgcatcgtgaa |
| ELP4-E5-F | attggaccagtatcatcttc | F6 | tgcctcccaaagtgctaaga |
| ELP4-E5-R | ttctactttgagagttgaag | R6 | gcacacctcttcctcatcct |
| ELP4-E6-F | ttctttgacccctggctaca | F7 | ccttgtgttttggcctctcc |
| ELP4-E6-R | ctgaggattggatccatcaa | R7 | ggaatgccattgtgtcgagt |
| ELP4-E7-F | aggaattcagaatcttggctcac | F8 | tgccattatttctgggatgtt |
| ELP4-E7-R | tggatcagatgtgttggcat | R8 | ggaattaatgtggtttgattcca |
| ELP4-E8-F | ttttcatgagatggcataggg | F9 | agctggccacttgataaggt |
| ELP4-E8-R | agcctgctcctgctcttctc | R9 | cttgaagaactggctgcctg |
| ELP4-E9-F | gattgattcatatacggcag | F10 | aagtgcccttccaagatgtg |
| ELP4-E9-R | ctcaatggtgaatagcttc | R10 | cagcaagatggcagtttcaa |
| ELP4-E10-F | gggtctccaaagtctctgct | F11 | tgggaatgtctttaacccaaa |
| ELP4-E10-R | ttgcagcctacattccctga | R11 | gggttgacttgacagcacct |
| F12 | tgtctcaaccaaaccaccct | F13 | aaggtgcccagcctaattct |
| R12 | gcgagagggagagagacttc | R13 | gtgtggtagcccactcctgt |
| F14 | ccatccatgctaggaggaaa |  |  |
| R14 | tgcacgtgagatgagtctcc |  |  |
