# Supplementary material for: A novel microdeletion of 517 kb downstream of the PAX6 gene in a Chinese family with congenital aniridia
Source: BMC Ophthalmol. 2023 Sep 26;23:393. doi: 10.1186/s12886-023-03147-1 (PMC10523764; doi:10.1186/s12886-023-03147-1)
Supplement: Supplementary file 5 — Additional file 5: Supplementary Table 3. [file 12886_2023_3147_MOESM5_ESM.docx]

| Supplementary Table 3 | References | [1] | [2] | [3] | [4] | [5] | [6] | [6] | [7] | [7] | [2] | [8] | [5] | [2] | [5] | [2] |  |
| --- | --- | --- | --- | --- | --- | --- | --- | --- | --- | --- | --- | --- | --- | --- | --- | --- | --- |
|  | Phenotype | aniridia | isolated aniridia | aniridia, autism, mental retardation | aniridia, developmental delay, a submucous cleft palate, ventriculoseptal defect | partial aniridia, ataxia, developmental delay | aniridia | aniridia | partial aniridia | aniridia | isolated aniridia | aniridia | aniridia | isolated aniridia | aniridia | isolated aniridia |  |
|  | Genomic coordinates  (GRCh38/hg38) | chr11:31,259,081–31,783,781 | chr11:31,626,701–31,782,540 | chr11:30,426,631–31,780,809 | chr11:31,728,036–31,777,121 | chr11:31,256,272–31,773,691 | ND | ND | chr11:31,584,312–31,762,042 | chr11:31,150,863–31,753,909 | chr11:30,720,065–31,753,909 | chr11:31,178,841–31,742,480 | chr11:31,400,877–31,729,876 | chr11:31,150,893–31,726,054 | chr11:30,988,877–31,725,876 | chr11:30,411,220–31,714,200 |  |
|  | Size of deletion | 525 kb | 155 kb | 1.3 Mb | 49 kb | 517 kb | 1,105 kb | 975 kb | 178 kb | 603 kb | 1,034 kb | 564 kb | 329 kb | 575 kb | 737 kb | 1,303 kb |  |
|  | Distance from  the 3′ of of *PAX6* | 1 kb | 2 kb | 4 kb | 8 kb | 11 kb | 11.6 kb | 22.1 kb | 23 kb | 31 kb | 31 kb | 46.5 kb | 54 kb | 59 kb | 59 kb | 70 kb |  |
|  | Deleted genes  (whole or partially) | *ELP4, IMMP1L, DNAJC24, DCDC1* | *ELP4* | *ELP4, IMMP1L, DNAJC24, DCDC1, MPPED2* | *ELP4* | *ELP4, IMMP1L, DNAJC24, DCDC1* | *ELP4, IMMP1L, DNAJC24, DCDC1* | *ELP4, IMMP1L, DNAJC24, DCDC1* | *ELP4* | *ELP4, IMMP1L, DNAJC24, DCDC1* | *ELP4, IMMP1L, DNAJC24, DCDC1* | *ELP4, IMMP1L, DNAJC24, DCDC1* | *ELP4, IMMP1L, DNAJC24* | *ELP4, IMMP1L, DNAJC24, DCDC1* | *ELP4, IMMP1L, DNAJC24, DCDC1* | *ELP4, IMMP1L, DNAJC24, DCDC1, MPPED2* |  |

| Table 3 (Continued) | References | [9] | [5] | [7] | [5] | [5] | [5] | [2] | [5] | [10] | [2] | [11] | [12] | **this study** | [13] | [14] | [15] |  |
| --- | --- | --- | --- | --- | --- | --- | --- | --- | --- | --- | --- | --- | --- | --- | --- | --- | --- | --- |
|  | Phenotype | partial aniridia | aniridia | rieger anomaly, aniridia | aniridia | aniridia | aniridia | isolated aniridia | aniridia | ocular coloboma | isolated aniridia | aniridia | aniridia | **aniridia** | aniridia | aniridia | aniridia |  |
|  | Genomic coordinates  (GRCh38/hg38) | chr11:31,100,614–31,699,482 | chr11:31,125,759–31,693,305 | chr11:31,096,480–31,689,028 | chr11:31,062,330–31,683,000 | chr11:30,896,519–31,676,709 | chr11:31,164,946–31,676,660 | chr11:30,899,512–31,675,137 | chr11:31,130,456–31,671,718 | chr11:30,989,367–31,670,690 | chr11:31,162,829–31,665,332 | chr11:31,096,280–31,662,139 | chr11:31,189,937–31,659,379 | **chr11:31,139,019−31,655,997** | chr11:31,307,764–31,650,108 | chr11:31,238,793−31,644,792 | chr11:31,091,031−31,322,068 |  |
|  | Size of deletion | 599–652 kb | 567 kb | 593 kb | 620 kb | 780 kb | 512 kb | 776 kb | 541 kb | 681 kb | 502 kb | 566 kb | 471 kb | **517 kb** | 350 kb | 406 kb | 231 kb |  |
|  | Distance from  the 3′ of of *PAX6* | 85 kb | 91 kb | 96 kb | 101 kb | 108 kb | 108 kb | 110 kb | 113 kb | 114 kb | 119 kb | 123 kb | 125.4 kb | **133 kb** | 135 kb | 140 kb | 467 kb |  |
|  | Deleted genes  (whole or partially) | *ELP4, IMMP1L, DNAJC24, DCDC1* | *ELP4, IMMP1L, DNAJC24, DCDC1* | *ELP4, IMMP1L, DNAJC24, DCDC1* | *ELP4, IMMP1L, DNAJC24, DCDC1* | *ELP4, IMMP1L, DNAJC24, DCDC1* | *ELP4, IMMP1L, DNAJC24, DCDC1* | *ELP4, IMMP1L, DNAJC24, DCDC1* | *ELP4, IMMP1L, DNAJC24, DCDC1* | *ELP4, IMMP1L, DNAJC24, DCDC1* | *ELP4, IMMP1L, DNAJC24, DCDC1* | *ELP4, IMMP1L, DNAJC24, DCDC1* | *ELP4, IMMP1L, DNAJC24, DCDC1* | ***ELP4, IMMP1L, DNAJC24, DCDC1 DCD1DCDC1*** | *ELP4, IMMP1L, DNAJC24, DCDC1* | *ELP4, IMMP1L, DNAJC24, DCDC1* | *DCDC1* | ND: Not done. |

References:

1. Zhang X, Zhang Q, Tong Y, Dai H, Zhao X, Bai F, Xu L, Li Y: **Large novel deletions detected in Chinese families with aniridia: correlation between genotype and phenotype**. *Molecular vision* 2011, **17**:548-557.

2. Plaisancié J, Tarilonte M, Ramos P, Jeanton-Scaramouche C, Gaston V, Dollfus H, Aguilera D, Kaplan J, Fares-Taie L, Blanco-Kelly F *et al*: **Implication of non-coding PAX6 mutations in aniridia**. *Human genetics* 2018, **137**(10):831-846.

3. Davis LK, Meyer KJ, Rudd DS, Librant AL, Epping EA, Sheffield VC, Wassink TH: **Pax6 3' deletion results in aniridia, autism and mental retardation**. *Human genetics* 2008, **123**(4):371-378.

4. Simioni M, Vieira TP, Sgardioli IC, Freitas EL, Rosenberg C, Maurer-Morelli CV, Lopes-Cendes I, Fett-Conte AC, Gil-da-Silva-Lopes VL: **Insertional translocation of 15q25-q26 into 11p13 and duplication at 8p23.1 characterized by high resolution arrays in a boy with congenital malformations and aniridia**. *American journal of medical genetics Part A* 2012, **158a**(11):2905-2910.

5. Blanco-Kelly F, Palomares M, Vallespín E, Villaverde C, Martín-Arenas R, Vélez-Monsalve C, Lorda-Sánchez I, Nevado J, Trujillo-Tiebas MJ, Lapunzina P *et al*: **Improving molecular diagnosis of aniridia and WAGR syndrome using customized targeted array-based CGH**. *PloS one* 2017, **12**(2):e0172363.

6. Lauderdale JD, Wilensky JS, Oliver ER, Walton DS, Glaser T: **3' deletions cause aniridia by preventing PAX6 gene expression**. *Proceedings of the National Academy of Sciences of the United States of America* 2000, **97**(25):13755-13759.

7. Addis L, Ahn JW, Dobson R, Dixit A, Ogilvie CM, Pinto D, Vaags AK, Coon H, Chaste P, Wilson S *et al*: **Microdeletions of ELP4 Are Associated with Language Impairment, Autism Spectrum Disorder, and Mental Retardation**. *Human mutation* 2015, **36**(9):842-850.

8. Syrimis A, Nicolaou N, Alexandrou A, Papaevripidou I, Nicolaou M, Loukianou E, Christophidou-Anastasiadou V, Malas S, Sismani C, Tanteles GA: **Aniridia due to a novel microdeletion affecting PAX6 regulatory enhancers: case report and review of the literature**. *Journal of genetics* 2018, **97**(2):555-562.

9. Wawrocka A, Budny B, Debicki S, Jamsheer A, Sowinska A, Krawczynski MR: **PAX6 3' deletion in a family with aniridia**. *Ophthalmic genetics* 2012, **33**(1):44-48.

10. Guo H, Dai L, Huang Y, Liao Q, Bai Y: **A large novel deletion downstream of PAX6 gene in a Chinese family with ocular coloboma**. *PloS one* 2013, **8**(12):e83073.

11. Cheng F, Song W, Kang Y, Yu S, Yuan H: **A 556 kb deletion in the downstream region of the PAX6 gene causes familial aniridia and other eye anomalies in a Chinese family**. *Molecular vision* 2011, **17**:448-455.

12. Liu X, Wu Y, Miao Z, Zhang H, Gong B, Zhu X, Huang L, Shi Y, Hao F, Ma S *et al*: **A novel deletion downstream of the PAX6 gene identified in a Chinese family with congenital aniridia**. *Ophthalmic genetics* 2018, **39**(4):428-436.

13. Vasilyeva TA, Marakhonov AV, Voskresenskaya AA, Kadyshev VV, Käsmann-Kellner B, Sukhanova NV, Katargina LA, Kutsev SI, Zinchenko RA: **Analysis of genotype-phenotype correlations in PAX6-associated aniridia**. *Journal of medical genetics* 2021, **58**(4):270-274.

14. Bayrakli F, Guney I, Bayri Y, Ercan-Sencicek AG, Ceyhan D, Cankaya T, Mason C, Bilguvar K, Bayrakli S, Mane SM *et al*: **A novel heterozygous deletion within the 3' region of the PAX6 gene causing isolated aniridia in a large family group**. *Journal of clinical neuroscience : official journal of the Neurosurgical Society of Australasia* 2009, **16**(12):1610-1614.

15. Wawrocka A, Walczak-Sztulpa J, Socha M, Kuszel L, Sowinska-Seidler A, Budny B, Bukowska-Olech E, Pilas-Pomykalska M, Jamsheer A, Krawczynski MR: **Homozygous microdeletion in the 11p13 region in the patient with isolated form of aniridia: New challenges in the genetic diagnostics of aniridia**. *American journal of medical genetics Part A* 2022, **188**(2):642-647.
